# Der Ergotismus – ein Ackerunkraut aus Mesopotamien wurde in Europa zum noch immer aktuellen Epidemie-Erreger

**DOI:** 10.1007/s10354-022-00960-z

**Published:** 2022-08-31

**Authors:** Heinz Flamm

**Affiliations:** 1 Martinstraße 7, 3400 Klosterneuburg, Österreich; 2 Medizinische Universität Wien, Wien, Österreich

**Keywords:** Ergotismus, Ergotalkoide, Claviceps purpurea, Ergotism, Ergotalcaloids, Claviceps purpurea

## Abstract

Der im Zwischenstromland in den Weizenäckern als Unkraut gewachsene Roggen wurde in Europa zur züchtbaren Nutzpflanze und in bestimmten Gegenden zur vorherrschenden Getreidesorte. Mit dem Roggen ist auch sein Parasit, der Mutterkornpilz Claviceps purpurea, nach Europa gekommen. Dieser Schlauchpilz infiziert einzelne Roggenkörner, worauf er das deren Größe übertreffende, die giftigen Ergotalkaloide enthaltende Sklerotium, das Mutterkorn, bildet. Diese Stoffe erzeugen die zwei charakteristischen Krankheitsformen, den Ergotismus gangraenosus und den Ergotismus convulsivus.

Das klinische Bild des Ersteren war bereits in der beginnenden Neuzeit und noch ohne Kenntnis der Ursache als „Ignis sacer“, „Antonius-Feuer“ und „Kalter Brand“ bezeichnet worden. Bei dieser fieberfreien Erkrankung meist der Extremitäten zersetzte sich die Muskulatur ohne Blutungen und ohne Schmerzen und bald brachen die muskelfreien Knochen ab. Früher oder später tat der Tod ein. Die frühere Therapie war die Amputation.

Der Ergotismus convulsivus, der meist in den deutschen Gebieten als „Kriebelkrankheit“ beschrieben wurde, begann mit dem Gefühl des Ameisenlaufens auf den Gliedern, gefolgt durch das charakteristische Symptom der sehr schmerzhaften Kontrakturen der Hände und Füße. Es kam auch zum Verlust der Sinne und der Sprache und schließlich zum Tod.

Die hauptsächliche Aufnahme des Pilzgiftes mit Roggenmehlprodukten, aber auch bei der Gewinnung und Bearbeitung des geernteten Roggens und auch die Frage von „Carry-over“ beim Genuss von tierischen Nahrungsmitteln und Milch werden besprochen. Es wird betont, dass zur Einhaltung der gesetzlichen österreichischen und EU-Höchstwerte an Sklerotien bzw. Ergotalkaloiden in gewissen für die menschliche und die tierische Ernährung bestimmten Getreideprodukten die geschilderten derzeitigen präventiven Maßnahmen vor dem Vermahlen weiterhin notwendig sind.

## Abstract (en)

In Mesopotamian wheat fields rye grew as weed and after certain developments rye reached Europe and there became a cultivable plant and the dominant grain in certain regions. With the rye also its parasite, the Claviceps purpurea, reached Europe. This ascomycete infects particular rye grains and in its developmental cycle grows to sclerotia bigger than each grain. These forms, the so-called ergots, contain the poisonous ergotalcaloids. After their intake they produce two characteristic forms of ergotism.

The clinical picture of the Ergotismus gangraenosus already before the knowledge of its origin, was known as “Ignis sacer”, “Anthony’s Fire” and “Cold Gangrene”. In this non-febrile affection mostly of the limbs, the muscles were decomposed without bleeding and without pain and thereafter the bones detached. Sooner or later the patients died.

The other form, Ergotismus convulsivus, mostly described in the German literature as “Kriebelkrankheit” (Crawly disease), begins with the sensation of running ants upon the limbs and continues with painful contractions of hands and feet. In many cases there is a loss of mind and language. Finally, death occurs.

The intake of ergotalcaloids predominantly with products of rye flour and meal but also during processing the harvested rye are discussed as well as the “Carry-over” by meat and milk. It is stressed that the compliance with the legal Austrian and EU regulations for the maximum contents of sclerotia and ergotalcaloids resp. in products for human and animal nourishments only can be guaranteed by continuing the technics of separating the sclerotia and their parts.

Die Geschichte des Ergotismus als einer von den Zeitgenossen wissenschaftlich erkannten und festgestellten Krankheit reicht nicht über das 16. Jahrhundert zurück, aus dessen letztem Dezennium die ersten ärztlichen Nachrichten über die Krankheit veröffentlicht worden sind [1]. Aus den vielen Berichten über Seuchen vom Altertum bis in die Gegenwart glaubten verschiedene Medizinhistoriker [1–6] auch manche dieser Ereignisse als mögliche oder sichere Mutterkorn-Vergiftungsepidemie bezeichnen zu können. Ein mehrfach zitiertes Beispiel aus früheren Jahrhunderten ist eine aus dem Jahre 922 aus Frankreich und Spanien überlieferte Massenerkrankung, der 40.000 Menschen erlegen sein sollen.

Auch heutzutage besteht die Gefahr der Gesundheitsgefährdung durch Ergotismus noch immer, wenn nicht die gesetzlichen Vorkehrungen konsequent durchgeführt werden. Darüber und über die Entstehung der Kenntnis des Ergotismus wird in der Folge berichtet.

۞Man schrieb das Jahr 1829. Im Norden Deutschlands, im Hannoverschen, ist einem strengen Winter ein nasskalter Sommer, „reich an Meteoren [*!!!*], Wolkenbrüchen und Ueberschwemmungen“, gefolgt. Es herrschten „Misswuchs, Theuerung und Hungersnoth“ und es wurden meistens die ärmeren Familien von einer epidemisch, aber oft auch nur sporadisch auftretenden Krankheit befallen. Vielfach wie folgendes Beispiel zeigt [7].

Eines Abends anfangs Oktober dieses Jahres stürzte eine 19jährige Frau aus einer sehr armen Schäferfamilie bei der Feldarbeit plötzlich „laut über heftige Schmerzen in beiden Beinen, besonders in den Unterschenkeln“, schreiend zu Boden. Bei der bis zu diesem Zeitpunkt gesunden Frau waren etwa seit der Zeit ihrer Pubertät immer wieder anfallsweise ziehende Schmerzen in den Extremitäten aufgetreten, die unter Unwohlsein einige Tage angehalten hatten. Die anfangs unter heftigen Schmerzen sehr unregelmäßig ablaufende Menstruation ist bald gänzlich ausgeblieben. Davon abgesehen, ist sie bis zu dem plötzlichen Ereignis gesund geblieben. Sie wurde nach dem Sturz vom Feld nach Hause getragen und ins Bett gelegt. Die heftigen Schmerzen steigerten sich und es wechselten Fieber und eisige Kälte (Frigor glacialis). Nach etwa 12 bis 14 Tagen „hatten sich unter Fortdauer der heftigen Schmerzen und der übrigen Erscheinungen, zuerst um die Malleoli beider Unterschenkel, weiße Flecken gebildet, von der Größe bis zu einem Thalerstücke“ [*Ø 3,8* *cm*]. Diese Flecken verfärbten sich dann livid, „während sich an anderen Stellen der Füße und der Unterschenkel bis vier Querfinger unterhalb der Kniegelenke immer mehr ähnliche Flecken gebildet hatten“, die später konfluierten. „Bis dahin hatte sich nur wenig Geschwulst eingestellt, und die Haut war an diesen Stellen immer trocken geblieben; endlich war die livide Farbe der Haut immer dunkler geworden, die Geschwulst hatte nach und nach, unter Fortdauer jener heftigen Schmerzen, zugenommen.“

Der mehrere Wochen nach Beginn der Erkrankung zugezogene Stadtwundarzt machte in die trockene und ganz schwarz gewordene Haut beider Unterschenkel mehrere kleine Einschnitte und begann kontinuierlich eine Räucherung mit Eichenrinden-Dekokt und Kamillenblüten-Aufguss. Während der Fortsetzung der Therapie war vier Querfinger breit unterhalb beider Kniegelenke eine Demarkationslinie entstanden, unterhalb der an den Inzisionsstellen konfluierende Geschwülste aus der Tiefe auftauchten. Diese brachen bald auf und entließen eine „missfarbene, ichoröse [*blutig-seröse, auch jauchige*] Flüssigkeit“. Binnen zwei bis drei Tagen waren ganze Muskelpartien abgestoßen worden, die gleichfalls ein dunkles, missfarbenes Ansehen hatten. Von der Demarkationslinie hingen jetzt „ganze Muskelpartien“ herab. Diese „hatten ein sehr dunkles missfarbiges Ansehen und soweit die Knochen entblößt waren, sahen sie erdfarbig, abgestorben und wie necrotisierte Knochen aus“. Schließlich brach nach einer kleinen Bewegung der linke Unterschenkel unterhalb der Wade ab (Abb. 1 und 2). Am folgenden Tag wurden operativ am anderen Bein von der Demarkationslinie aus die gesunden Muskelpartien etwa 2 Zoll lang von den Knochen getrennt und mit einem Retraktor zurückgezogen, worauf der Knochen, so hoch wie es möglich war, abgesägt wurde. Die Blutgefäße mussten nicht abgebunden werden. „Ueberhaupt erfolgte auch während des Verlaufs der Krankheit keine Blutung.“ Unter guter Wundversorgung und Fürsorge vernarbten die Wunden und die Knochenstümpfe bedeckten sich mit „guten Fleischpolstern“. Nach einem halben Jahr war der Allgemeinzustand besser als vor der Krankheit, doch führte plötzlich eine Apoplexie zu geistigen Einschränkungen und Verlust der Sprache. Eine weitere Apoplexie verursachte ein Vierteljahr später den Tod der jungen Frau.

**Figure 1:**
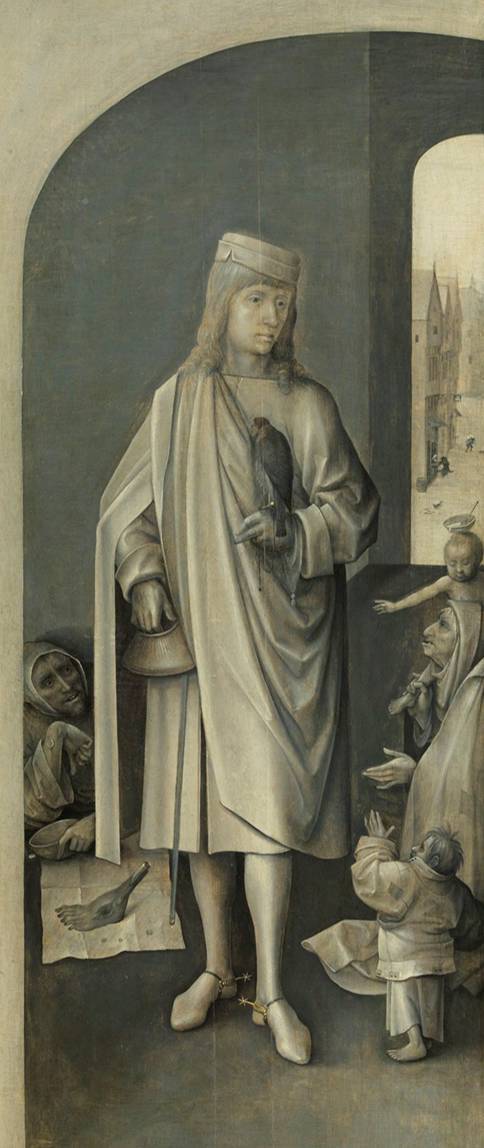

**Figure 2:**
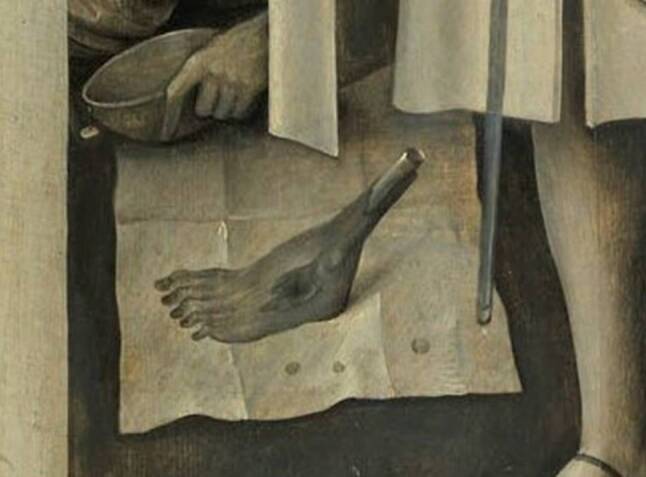

Bei manchen Kranken konnte die Gangrän alle äußerlich sichtbaren Organe erfassen. Es sind Fälle beschrieben, bei denen die von der Krankheit Befallenen alle vier Extremitäten verloren haben, was sie bei guter Pflege noch einige Tage überleben konnten [8]. Es verloren manche Kranke durch die Gangrän auch Nasenspitzen, Skrotum [9] oder Brüste [3], wahrscheinlich auch die Ohren. Auch Grauer Star wird berichtet [10].

Eine in der Literatur geschilderte fürchterliche Erkrankung einer ganzen Familie gibt Sievers wieder [7]: Die Eltern und sechs Kinder klagten über heftige Schmerzen, die nur die unteren Extremitäten ergriffen; ein 4 Monate alter Säugling starb nach Abfall seiner Glieder, die Mutter und die anderen fünf Kinder verloren insgesamt sieben Unterschenkel und weitere vier einzelne Füße, also insgesamt elf Füße der sechs Personen, der Vater blieb unversehrt.

Sievers zitiert auch einen anderen schrecklichen Fall: Bei einem Bauern führte die Gangrän dazu, dass ihm „anfänglich alle Zehen, darauf beide Füsse abfielen, und endlich sich das Fleisch von beiden Unter- und Oberschenkeln trennte, und nichts weiter als die Knochen dieser Theile zurückliess“.

Nicht bei allen von der Krankheit Befallenen ist es zur vollen Ausbildung sich ausdehnender Gangrän gekommen. Bei manchen Kranken kam es bevor die Gangrän richtig entstand zu Symptomen, die im Abschnitt bei der Kriebelkrankheit beschrieben werden.

## Ignis sacer – das Heilige oder eher das Verfluche Feuer

Für die fürchterliche, sehr oft tödliche Krankheit benötigte man Namen. Die mittelalterlichen Ärzte wusste aus den alten Schriften, dass die Römer mit „Ignis sacer“, das ist „Heiliges“, aber auch „Verfluchtes Feuer“, alle möglichen Veränderungen auf der Körperoberfläche bezeichneten, die heftiges Brennen verursachten, um sich griffen und andere den Wirkungen des Feuers analoge Symptome zeigten.

Dieser Begriff wurde vom 10. bis 12. Jahrhundert in verschiedenen Ländern Europas, vorwiegend in Frankreich, den Niederlanden und Lothringen, für schwere Leiden mit Absterben von Gliedmaßen wiederverwendet. Die Ärzte sprachen vom Ignis sacer, Feu sacré, Mal des ardens [*Brennendes Leiden*] oder Pruna [*Dörrpflaumen*] [11], bald aber änderte der Begriff seine Zuordnung von den antiken Göttern zum christlichen Glauben als Ignis Sancti Antonii. Im 12. Jahrhundert wurde der Glaube verbreitet, dass vom Heiligen Feuer betroffene durch Wallfahrt zur Begräbnisstätte des Heiligen Antonius, des Einsiedlers, in Vienne in der Dauphiné geheilt wurden [12]. In der Folge bildeten sich noch weitere z. T. religiöse Bezeichnungen wie Ignis Beatae Virginis, Ignis Sancti Martialis [*wahrscheinlich nach Martius von Clermont* *=* *M. v. Auvergne*], (St.-)Antonius-Rache, (St.-)Antonius-Plage, Muttergottes-Brand oder nur lokal übliche Bezeichnungen wie Mutterkorn-Brand oder Ziehende Seuche [13].

Der Heilige Antonius, der Einsiedler, ist zum Namenspatron des Heiligen Feuers geworden, nachdem sich die Antonius-Bruderschaft um die am Ignis sacer Erkrankten angenommen hatte. Diese Laiengemeinschaft war 1095, zu Zeiten des Wütens des Heiligen Feuers, in La Motte-aux-Bois [*heute: Saint-Antoine*] in der Dauphiné gegründet worden. Sie begann mit einem Hospital für Kranke und Pilger, dem ein „Spital für Verstümmelte“ und bis Ende des 12. Jahrhunderts lokale Hospize in Italien, Spanien und den deutschen Ländern folgten. Die Bruderschaft wurde 1202 vom Pabst zu einem Augustiner-Chorherrenorden erhoben. Der Hl. Antonius erhielt durch die Tätigkeit der Antoniter seine Attribute: die Glöckchen zum Aufruf für Spenden, das Schwein als Objekt der Spenden und seinen Wanderstock, dessen oberes Ende als Symbol für eine Krücke ein mit Glöckchen behängtes Τ [*griech. „Tau“*] bildete, das auch seine Kleidung zierte; die Schweine trugen z. T. auch Glöckchen zur Kennzeichnung als dem Antoniter-Hospital gehörend [5, 14–16].

Ignis sacer und seine Synonyme kamen im deutschen Bereich etwa im 17. Jahrhundert aus dem allgemeinen Gebrauch. Man verwendete den schon seit dem Mittelalter gleichgesetzten Begriff „Brunst“ [*Feuersbrunst*], „Prant“ oder „Brand“ für verschiedene Schäden der Gewebe bis zu deren Zerstörung. Eine ausgezeichnete Erklärung aller zu seiner Zeit bekannten Fakten lieferte Wilhelm Fabry von Hilden (= Gulielmus Fabricius Hildanus, 1560–1634) im Jahre 1593 in seiner danach noch zehnmal neu aufgelegten berühmten Schrift „De gangraena et sphacelo. Das ist: Von dem heissen und kalten Brandt, oder S. Antoni und Martialis Fewer“ [17]. Der Begriff „Sphacelus“ wurde von manchen Autoren synonym mit Gangraena verwendet, z. B. von Rudolf Jakob Camerarius (1665–1721) für eine letal beendete Gangrän der Füße eines Bauern aus dem Schwarzwald [18].

Fabry von Hilden schrieb vom „Heißen Brand“, wenn an einer Gliedmaße „ein Zustand begonnen hat“, der „gewöhnlich durch starke Hitze, überaus große Schmerzen, Schwellung und andere hitzige Erscheinungen gekennzeichnet ist, und weil solche Hitze, Schwellung usw. wie ein brennendes Feuer um sich greift und sich nach allen Orten ausbreitet“. „Wenn nun aber solche Hitze die eingepflanzte Wärme, das Blut und die natürliche Feuchtigkeit aufgebraucht hat“, nennt man diesen Schaden seit Hippokrates und Galen „Sphacelus“. „Weil bei diesem die befallene Stelle schwarz und eiskalt ist, die Krankheit aber gleichwohl (wegen der Fäulnis) noch fortschreitet und die Nachbarschaft angreift“, will Fabry von Hilden sie „mit etlichen vortrefflichen gelehrten Ärzten <kalten Brand> nennen“.

Wichtig ist, dass der Heiße Brand nur Haut, Muskeln und andere Weichteile betrifft, der Kalte Brand daneben auch die Knochen, die auch abbrechen können. „Im Heißen Brand ist die Haut noch rötlich und heiß, gleich wie brennende Glut und Kohle, dagegen ist die Haut im Kalten Brand aschfarben und schwarz, gleich wie ausgebrannte und erloschenen Kohlen“ und die ganze Stelle ist „faul und riecht überaus übel“. Beim Heißen Brand spürt der Kranke den Stich mit einem spitzen Instrument in den erkrankten Teil, beim Kalten Brand dagegen nicht. Die Behandlung ist beim Heißen Brand darauf zu richten, dass die Fäulnis nicht eintritt und, wenn diese bereits besteht, dass sie nicht fortschreitet und sich festsetzt. Beim Kalten Brand muss „das Faule und Verdorbene“ durch Schneiden, Ätzen oder Brennen entfernt werden.

**Figure 3:**
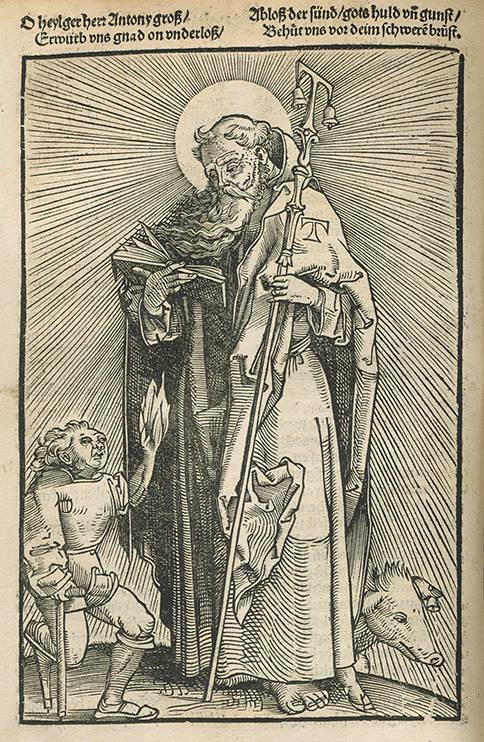

Mit der Erkennung, Unterscheidung und Behandlung des Heißen und Kalten Brandes setzte sich bereits 1517 der sehr erfahrene Straßburger Wundarzt Hans von Gersdorff (2. Hälfte 15. Jhdt.–Anfang 16. Jhdt.) in seinem von internationalen Zeitgenossen geschätzten und noch heute bekannten „Feldtbuch der Wund-Artzney“ auseinander. Darin bezieht er sich im Text, unterstützt durch einen Holzschnitt (Abb. 3), auch auf das Antonius-Feuer. [19].

Noch bis heute ist die Frage offen, welche Krankheiten und Seuchen man unter den Bezeichnungen „Ignis sacer“ und „Brand“ subsumieren soll. Medizinhistoriker bemühten sich, in den überlieferten Berichten über Epidemien vom griechisch-römischen Altertum bis in ihre Gegenwart derartige Erkrankungen nachzuweisen. Die Ergebnisse sind mehr oder weniger lange Listen mit Berichten über verschiedene epidemische Krankheiten [*wahrscheinlich u.* *a. Erysipel, Pest, Anthrax, Zoster, akute Exantheme, Pocken, Skorbut*], deren Natur aus den Beschreibungen nur unsicher zu deuten ist [1, 3, 5, 20, 21].

Im 17. Jahrhundert erwachte in Frankreich das Interesse an dieser dort epidemisch auftretenden Krankheit [11, 22, 23]. So berichtete im Jahre 1676 Claude Perrault (1613–1688) der Academie royale des sciences (Paris), er habe bei einem Besuch in der Sologne von Ärzten und Wundärzten erfahren, dass nach Genuss von aus verdorbenem Roggen erzeugtem Brot Gangränfälle aufgetreten sind. In deren Verlauf ist es zum Abfallen von Fingern und Zehen, auch von ganzen Händen und Füßen oder von Nasen und anderen Körperteilen gekommen. Den Prozessen sind weder Fieber, noch Entzündungen oder wesentliche Schmerzen vorausgegangen. Auch sein Zeitgenosse Claude Bourdelin (1621–1711) hatte gleich Fälle zwei Jahre zuvor in Montargis beobachtet.

Solche Berichte waren offenbar der Anlass, dass Denis Dodart (1624–1707) von der Pariser Akademie beauftragt wurde, Untersuchungen über diese Erscheinungen anzustellen. Als Ergebnis vieler Befragungen konnte Dodart 1676 berichten, dass er als vermutliche Ursache entarteten Roggen in Bereichen von Sologne, Berry, Gastinois und den Pays Blaisois und besonders in den Gegenden mit lockeren und sandigen Böden gefunden hat. Es war ihm über viele Jahre berichtet worden, in denen das Getreide schlecht gewesen ist. Solches ereignete sich stets nach regnerischen Frühjahren mit folgenden nassen, heißen Sommern. Die Folgen waren das Auftreten von Fällen malignen Fiebers mit Schmerzen und Fantasieren und dem Aufhören der Milchproduktion der Frauen. Eine sich entwickelnde Gangrän betraf gewöhnlich als erstes die Extremitäten. Diese Krankheit glich diesbezüglich überhaupt dem damals schon bekannten Skorbut gewisser Bevölkerungen [22, 24]. Die Frage „Ob die Kriebelkranckheyt Gemeinschafft habe mit dem Schorbock [*Skorbut*]/vnnd wie sie curiret werden müsse?“ hatte sich schon 1615 Gregor Horst (1578–1636) in seinem 1615 erschienenen „Büchlein von dem Schorbock [*Skorbut*]/Gemeynem Vatterlandt zum besten Teutsch beschrieben“ gestellt, in dem er auch zur Behandlung der Kriebelkrankheit „Antidotum convulsivum oder KriebelTheriack“ und „Pulvis convulsivus oder Kriebelpulffer“ empfohlen hat [25]. [*Der Theriak galt vom Altertum bis ins 18. Jahrhundert als wichtiges, äußerlich und innerlich anzuwendendes, vorbeugendes und heilendes Mittel; er wurde nach verschiedenen Rezepturen aus bis zu 61 Stoffen (z.* *B. Bezoar, Ambra, Perlen, Edelsteine, Korallen, Terra sigillata, Perubalsam, Hirschhornsalz, Weinstein, versch. Pflanzen, Latwergen) gemischt und musste vor der Anwendung einige Zeit reifen*.].

Dodart referierte 1676 auch einen schriftlichen Bericht seines Kollegen Thuillier über Beobachtungen von dessen Vater bei der armen Bevölkerung der Campagne während des unheilvollen Jahres 1630. Damals gelangten ein Arzt und ein Wundarzt zur Meinung, der von Mutterkorn befallene Roggen (Seigle cornu) sei die Ursache der damals sehr häufigen Gangränfälle. Von den Jahren 1650, 1670 und 1674, die alle feucht und stürmisch waren, sind weitere Ausbrüche, auch in anderen französischen Departements, bekannt geworden [22].

Hundert Jahre danach (1776) ließ die französische Société Royale de Médecine die als St.-Antonius-Feuer bezeichnete Erkrankung durch eine aus den Herren de Jussieu, Paulet, Saillant und Tessier gebildete Kommission untersuchen [11]. In der Sitzung vom 31. Dezember 1776 wurden vorwiegend aufgrund der Berichte aus dem 18. Jahrhundert die verschieden intensiven Krankheitsbilder und die damals angewandten Therapien sehr eindringlich beschrieben. Als Ergebnis der Untersuchungen berichtete die Kommission die Erfahrungen und Beobachtungen der Herren Dodart, Lang, de Salerne, Duhamel, Arnaud de Nobleville, Réad und anderer nicht Genannter, dass ohne Zweifel das St.-Antonius-Feuer „sur les effets de l’ergot du seigle“ [*durch die Wirkung des Sporns von Roggen*] erzeugt wird. Es wurde auch ein gegenteiliger Bericht von Camerarius [18] über diese Gangrän der Extremitäten bei Personen referiert, die sicherlich nicht Mutterkorn gegessen haben, und auch, dass in Thüringen die Hebammen Getreide mit Mutterkorn [*dort „Rockenmutterle“ genannt*, [18]] zur Förderung der Entbindung verwenden.

Wegen solcher und weiterer in der Literatur zu findender Widersprüche wollten die Mitglieder der Kommission ihre Meinung vorerst suspendieren, bis mehr Fakten mitgeteilt werden, sodass eine Entscheidung möglich sein wird.

**Figure 4:**
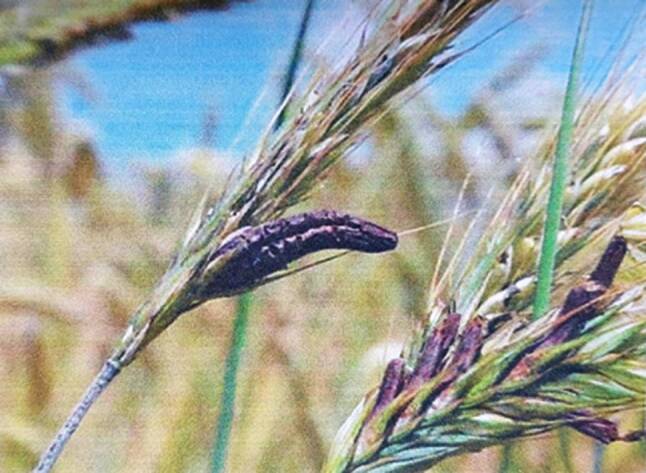

Die französischen Begriffe „Ergot du seigle“ und auch „Seigle cornu“ [*gehörnter Roggen*] beschreiben das Aussehen des Mutterkorns, das wie ein Sporn [*ergot*] oder Horn [corne] aus der Roggenähre hinausragt (Abb. 4, [26]). So ist „Ergotismus“ zuerst im französischen Bereich zum Synonym von St.-Antonius-Feuer und den analogen „Feuer“-Begriffen geworden und wurde damit vorerst nur für die oben beschriebene gangränöse Erkrankung verwendet.

## Die Kriebelkrankheit

Im April 1597 berichteten die „Professores Facultatis Medicæ der Universitet zu Marpurg in Hessen“ „Von einer vngewöhnlichen/vnnd biß anhero in diesen Landen vnbekannten/gifftigen/ansteckenden Schwacheit/welche der gemeyne Mann dieser orte in Hessen/die Kribelkranckheit/Krimpffsucht/oder ziehende Seuche nennet“ [27]. Auf 33 Seiten beschrieben sie die Krankheit, die „an sich selbsten aber nichts anders ist/als eine agitatio cõvulsiva, oder Krampffsüchtige regung vnd ziehen der glieder/so sich von gifftiger vnd ansteckenden qualitet in hånden vnd fůssen anfengt//vnd von dannen fort wandert/vnd den gantzen Leib zeucht/convellirt [*zerreißt, erschüttert*], vnd endlich auch in die schwere Plag/Toll vnd Taubsucht/vnd viel andere beschwerlichen affecten zu verwechßlen pflegt“.

In den folgenden zwei Jahren hat sich weiterhin die bisher „dieser orten vnbekannte/vnd gantz vngewöhnliche convulsio“ „deromassen außbreitet/daß“ sie in Hessen und manchen anderen Orten, „(in welchen diese schwacheit schon etliche jahr gehalten)“ „die leuth darinn elendiglich zurichtet vnd verderbet“. Das „Tractåtlein“ der Professoren sollte „nur allein zu forderung [*Förderung*] der armsten vñ angefochtenen leuthe/welche diese schwacheit viel mehr vnd hefftiger/als die wohlhabende plaget“ dienen.

„Diese Kribelkranckheit ist anders nichts/als eine gifftige/ansteckende intếperies [*Übermaß*]/der Nerven vñ Sehnader [*?*] deß leibs/so von gifftigen schleimen vnd dåmpffen entstehet/vnd nach vnderscheid der complexiorum [*Zusammenwirken*]/mit vnnatůrlichem zůcken vñ lauffen/auch convulsionibus der glieder/der schweren noth/tieffen schlåffen/wahnwitz/auch etwa vnsinnigem wůten vnd rasen/bißweilen mit/zuzeiten auch ohne Feber vnnd hitz/[*je*] nachdem sie die Complexion der Patienten disponirt befindet/sich erzeiget vnd hervorbricht“.

Schon vor dem Bericht der Professoren, zumindest um 1577 [28], ist diese Krankheit von vielen Orten Deutschlands (Lüneburg, Westfalen, Köln, Hessen, Coburg, Sachsen, Breisgau), von Schlesien und der Schweiz (Zürich, Luzern) berichtet worden. In den verschiedenen Gegenden nannte sie das Volk Kriebel- oder Hiebelkrankheit, auch Krampfsucht oder epidemische Epilepsie [2]. In den medizinischen deutschen und lateinischen Publikationen wurde sie anfangs als „Raphania“ bezeichnet (z. B. [21, 28–31]). Dieser Namen stammte vom berühmten schwedischen Arzt und Botaniker Carl von Linné (1707–1778), der ihn 1763 vom Ackerunkraut Raphanus raphanistrum [*Hederich, Ackersenf, Ackerrettich, Wilder Rettich*] abgeleitet hat, dessen länglichovale Samen er als Verursacher der Kriebelkrankheit gehalten hat [32].

Auf Dauer hat sich aber nicht „Raphania“, sondern „Kriebelkrankheit“ durchgesetzt. In den lateinischen Publikationen war dies der „Morbus cerealis convulsivus“ oder „Morbus cerealis spasmodicus“ im Gegensatz zum „Morbus cerealis gangraenosus“ [„*Gangraena epidemica“* [33]] für die primär französische Bezeichnung „Ergotismus“. Über die geographische Verbreitung der beiden Formen schrieb der berühmte Medizinhistoriker August Hirsch (1817–1894) im Jahre 1860, „dass der Ergotismus, innerhalb der neueren und neuesten Zeit, in Frankreich, Spanien und der Schweiz fast immer in Form des Ergotismus gangraenosus (Ignis sacer), dagegen in Deutschland, Holland, Italien, Schweden, Norwegen, Russland und Finnland fast ausschliesslich als Ergotismus convulsivus aufgetreten ist“.

Zur genauen Kenntnis der Kriebelkrankheit sei der Bericht aus Norddeutschland des 18. Jahrhunderts von Johann Ernst Wichmann (1740–1802) zitiert [6]. Unter gleichen Wetterverhältnissen wie für den Ergotismus in Frankreich angegeben brach Ende August 1770 in vielen Dörfern Niedersachsens in vorwiegend den ärmeren Bevölkerungsgruppen plötzlich eine andersartige Massenerkrankung aus. Die Befallenen gebärdeten sich unter äußersten Schmerzen und heftigen Krämpfen „unsinnig“. „Diese bösartige Krankheit dauerte, mit abwechselnden Vermehrungen und Verminderungen der Zahl der Kranken, bis in den März. Nach dem Schlusse dieses Monaths befielen keine mehr. Aber bey den schon krank gewesenen, stellten sich desto öftere Rückfälle zu ungewissen Zeiten ein“. Viele starben schon zu Anfang der Erkrankungen bei den ersten Krämpfen. Aber auch noch im folgenden Sommer erlagen manche den Folgen der Krankheit. Durch die Todesfälle sind manche Häuser gänzlich ausgestorben.

Nach Gerhard Matthias Friederich Brawe (1745–1787) in Verden, Niedersachsen, brach die Krankheit bei manchen Dorfbewohnern ohne Vorzeichen aus. Andere verspürten „etliche Tage vor einem deutlichen Anfang ihrer Krankheit einige Müdigkeit und Schwere in den Gliedern, die Eßlust war weg, der Schlaf unruhig und der Kopf etwas schwindlich und schwer. Bei andern hingegen ließ sich vorher im Cörper nichts kränkliches wahrnehmen“, bis plötzlich, besonders in der Nacht und bei vermehrter Wärme, ganz gelinde Zuckungen von einzelnen kleinen Muskelfasern bis von ganzen Muskeln (wie der M. orbicularis oris) auftraten, was der Beobachter sehen und mit den Fingern tasten konnte. Diese Krämpfe steigerten sich bei jeder Wiederkehr. Dazu kamen Empfindungen als krabbelnden Ameisen über Arme, Beine und Gesicht. Es erfolgte hierauf ein Zucken in Händen und Füßen, mit heftigen Schmerzen, die sich von den Zehen bis ins Knie, und von den Fingerspitzen bis in den Ellenbogen erstreckten; alsbald wurden die Hände und Füße krumm zusammengezogen, und die Kranken konnten, ohne die heftigsten Schmerzen zu empfinden, kein Glied selbst bewegen. „Die Hände und Füße litten auch ein starkes Brennen, und manchmal war solches mit einem sehr starken Schweiß vergesellschaftet. Der Kopf war dabey sehr düster und schwer, sie empfanden auch darin starke Schmerzen; sie sahen vor den Augen goldene Kügelchen sich bewegen, und manchen kam es vor, als wenn ein Flor vor ihren Augen ausgebreitet wäre. Einige bekamen starke Krämpfe im Unterleibe, bey andern, und besonders bey Kindern, erfolgten auch öftere Anfälle von Zückungen in dem ganzen Cörper. Etliche empfanden eine Uebelkeit, Neigung zum Erbrechen, und würkliches Erbrechen. Einige klagten über Bitterkeit des Geschmacks, nebst Uebelkeit, ohne sich zu erbrechen. Bey einigen war der Leib einige Tage verschlossen; andere hatten zwar natürliche aber sehr beschwerliche Leibesöffnung [*Stuhlgang*]; bey denen aber, welchen diese gut von statten ging, waren die Anfälle jederzeit gelinder. Oft endigten sich die Anfälle mit einem starken Schweiß, der aber doch nie die Krankheit aufs zukünftige verminderte. Die mehreste Zeit mangelte bey diesen Kranken der Schlaf. Hände und Füße blieben anfangs fast beständig, auch ausser den stärksten Anfällen, etwas zusammengezogen, und wenn man sie ausdehnen wollte, so zogen sie sich gleich wieder krum, doch schien es denen Kranken einige Erleichterung zu verschaffen, wenn man diese Theile oft ausdehnte. Die Anfälle hielten niemals eine gewisse Zeit und Ordnung, und dauerten bald kürzere, bald längere Zeit. Oft kamen etwan in 24 h 3, 4, 6, ja 8 Anfälle, oft aber auch wenige, und zuweilen noch mehrere. Sie hielten zu Zeiten 2, 3 und mehrere Stunden an, und gingen selten innerhalb einer Stunde wieder vorüber. Besonders aber waren bey denen Kindern vom 1ten bis zum 7ten Jahre Convulsionen sehr gewöhnlich, und machten oft in 24 h gegen 6 starke Anfälle. Bey allen äusserte sich nach dem Anfall eine sehr starke Entkräftung, die Kranken taumelten wie Betrunkene, ihr Kopf war ihnen sehr schwindlich, und die wenigsten waren des Gebrauchs ihres Verstandes völlig mächtig. Alle hatten aber doch einen sehr starken Hunger, und konnten viele Speisen, ohne Beschwerden zu sich nehmen. Magendrücken empfand keiner von ihnen, und nach den Anfällen glaubten sie, die gemeldete Mattigkeit ausgenommen, völlig gesunde Leute zu seyn. Im Puls war auch nichts widernatürliches anzutreffen, und vom ungewöhnlichen Durst waren sie ebenfalls ganz frey. Diese Zufälle [*Zustände*] wurden mehrentheils alle zusammen bey jedem Kranken bemerket“ [34].

Das charakteristische Gefühl des Krabbelns von Ameisen, der „Sensus formicationis“, auch „Myrmezismus“ oder „Myrmeciasis“ genannt [*Formica* *=* *Ameise, Myrmecia* *=* *ameisenähnliche Spinnentiere*], gab der Krankheit den Namen „Kriebelkrankheit“.

Bei der „bösen Art“ der Kriebelkrankheit mit kurzem Verlauf empfanden die Kranken „noch kurz vorher nichts, auch kein Ameisenlaufen oder Kriebeln. Es überfällt sie auf einmal Blindheit und Schwindel, welcher sie zu Boden wirft, sie ihrer Sinne gänzlich oder zum Theil beraubt, Zittern der Glieder, heftiges aber vergebliches Würgen verursacht, starcke Zuckungen macht, und zugleich alle Gelenke krampficht zusammenzieht, und einwärts beugt; so, daß gemeiniglich alsdenn die Ellenbogen gegen die Brust gedrückt sind. Das Handgelenk ist noch mehr gekrümmet und alle Finger sind in eine geballte Faust geklemmt. Die Gewalt der biegenden Muskeln der Arme und Finger ist dabey so stark, daß oft zween starke Männer sie nicht gerade zu machen oder aufzubrechen vermögen. Die Beine müssen ein gleichmässiges Zusammenziehen leiden. Die Fersen werden aufwärts gegen die grosse Achilles-Sehne getrieben und die Zähen unter die Fußsohle gepreßt.“ Die mit kaltem Schweiß bedeckten Kranken wälzen sich mit enormer Unruhe auf ihrem Lager und das gelbliche, eingefallene Gesicht ist oft von blutigem Schleim bedeckt. „Sie schreyen alle beständig um die Ausdehnung der Finger und Gelenke, und versichern unter unaufhörlichem Winseln, die davon entstehende Erleichterung.“ In den kurzen Intervallen „beklagen sie sich unaufhörlich über unaussprechliche Schmerzen, Drücken und Beklemmungen der Herzgrube, welche mit beständigem Würgen und fruchtlosem Erbrechen vergesellschaftet sind.“ Der Puls „bleibt klein, langsam, unterbrochen, und ist oft, wenn die Krämpfe recht grausam und wütend ansetzen, kaum zu entdecken“. Nachdem solche „traurigen und schreckhaften Auftritte“ mit sehr wenigen ruhigen Zwischenzeiten oft 24 und mehr Stunden angehalten haben, „melden sich wahre Zuckungen“ „mit allmähligem Verlust der Sinne und Sprache, und bei öfterer Wiederkehr derselben, endiget sich diese fürchterliche Vergiftung, nicht selten erst am dritten Tage, mit dem Tode“. Von dieser heftigen Art der Krampfsucht ist nach den Erfahrungen von Daniel Johann Taube (1727–1799) „noch kein einziger Kranker genesen“ [6].

In der Literatur findet man Berichte über kriebelkranke stillende Frauen. Wichmann hat „viele säugende Mütter gesehen, und zwar einige derselben im äussersten Grade der Krankheit, die davon ihren Säuglingen nichts mitgetheilet, auch verringerte diese Krankheit die Milch in den Brüsten nicht bey denen, welche das Kind bis auf die letzte Zeit des Lebens an der Brust behielten“ [8]. Auch Taube behandelte eine Frau, die bis zum Tode „völlige Milch“ behielt und deren Kind er „ein Jahr darauf völlig gesund und von der Krankheit frey geblieben“ sah. Weitere drei Fälle in lokalen Lazaretten waren ihm bekannt [6]. Dem gegenüber berichteten Dodart [22] aus Frankreich und Camerarius [18] aus Thüringen über das Aufhören der Milchproduktion bei stillenden Frauen mit Gangrän, die offenbar auch die Milchdrüsen erfasst hat.

In seinem Lehrbuch „Ueber die Erkenntniss und Cur der chronischen Krankheiten des Menschen“ aus 1817 [28] erklärte Wilhelm Andreas Haase (1784–1837) als charakteristisch für die von manchen Ärzten als chronisch bezeichnete Form der Kriebelkrankheit, dass es nach dem üblichen Beginn zu einer „deutlichen Exacerbation“ kommt, in deren Verlauf von mehreren Wochen verstärkte Paroxismen und vergleichsweise dazu mildere Remissionen einander abwechseln. Der Tod kann „entweder durch Brand oder durch Apoplexie“ eintreten.

Die fast gleichzeitig (1814) geschriebenen Wiener Dissertatio inauguralis medica von Philipp Budetz aus Horaschdowitz in Böhmen [21] bringt eine genauere Zusammenfassung der verschiedenen Ausgänge der Erkrankung.

Der Kranke kann demnach genesen, wenn er eine gute Konstitution besitzt und die Krankheit langsam beginnt, leicht ist und einen mehr chronischen Verlauf nimmt und wenn dem Kranken von Anfang an tatkräftige Hilfen geleistet werden.

Die Natur könne auch das Böse überwinden und die eingetragenen Gifte spontan auslöschen, wenn diese weniger „grässlich“ sind. Wenn die Anstrengungen der Natur in der akuten Krankheit und dem zuweilen beobachteten Fieber unzureichend sind, kann sich die Heilung auch unter Abgang von Spul- und Peitschenwürmern mit psorisiformen Exanthemen oder Hautabszessen unter verschiedenen Schmerzen und Schwitzen einstellen. Die Ärzte müssen aber bei den auftretenden Neurosen meistens die Natur unterstützen.

Die ehemalige Gesundheit kann nach 14 Tagen, nach einigen Wochen oder auch erst nach einigen Monaten zurückkommen, „was von der Intensität und Beschaffenheit des Übels, von der Stärke und Wirksamkeit der Krankheitsursache, von der Härte der Epidemie und von diversen bisher unbekannten Umständen abhängt“. Wiederholte Schlaflosigkeit lässt das Übel wieder aufflackern und pflegt leicht in Epilepsie umzuschlagen.

Nach einem langen Intervall in Gesundheit kann die Kriebelkrankheit als Rezidiv in der vorherigen oder einer geringeren Stärke wieder auftreten, sie kann aber auch in eine andere Krankheit umschlagen, und zwar in Hydrops [*Wassersucht*], in chronische Diarrhoea [*Durchfall*], in Hämatopoea [*Blutauswurf]*, in Tabes [*Schwindsucht*], in verschiedene nervöse Leiden, in Syringismus [*Ohrensausen*], Amblyopia [*Schwachsichtigkeit*], Amaurosis [*Blindheit*], Cataracta [*Grauer Star*], Vertigo [*Schwindel*], Dysmnesia [*Gedächtnisstörung*] oder totale Amnesia [*Erinnerungsverlust*], Fatuitas [„*Blödsinn“*] und Amentia [*Wahnsinn*], in Tetanus [*Krampf*] oder schwerst heilbare Epilepsie [*Fallsucht*], in Apoplexia [*Schlaganfall*], in Pareses und Paralyses [*leichte und komplette Lähmungen*] der Extremitäten.

Dem Tod gehen Prodrome voraus, die den Ausgang anzeigen. Das sind vor allem Krämpfe, Sprachverlust, allgemeine Lähmungen und Gangrän verschiedener Körperteile. Der Tod kommt häufig mit Schlaganfällen und Krämpfen.

Die an der Kriebelkrankheit Gestorbenen zeigen bei der Obduktion rasche Fäulnis, Zeichen septischer Entzündung und gangränöse Flecken verschiedener Organe, vorwiegend in Gehirn und Baucheingeweiden, sowie Extravasate in den Hirnventrikeln und in der Bauchhöhle.

Den Unterschied in der Entstehung von gangränöser Erkrankung und Kriebelkrankheit erklärte Heinrich Sievers [7] in seiner Inaugural-Abhandlung (1835) dadurch, dass bei Ersterer offenbar durch eine Überreizung der Nerven eine Paralyse auftritt, die sich dem Gefäßsystem mitteilt und so den Brand verursacht. Die konvulsive Form entstehe dagegen durch „Nervenaufreizung ohne Theilnahme des Gefässsystems“.

Im 17. Jahrhundert war der Glaube an üblen Zauber und Teufelswerke im Volk weitverbreitet, sodass arme Kranke, die als Folge ihrer Mutterkornvergiftung die Menschen durch Auftreten von Krämpfen und geistig-psychischen Symptomen beunruhigten, als verhext angesehen wurden. Den der Verhexung Beschuldigten wurden Prozesse gemacht. Die genaue Beurteilung der erhaltenen schriftlichen Unterlagen solcher Hexenprozesse, insbesondere deren Bezug zu Ergotismus ist sicher schwierig. Es gibt wissenschaftliche Aufarbeitungen für solche Prozesse in Massachusetts und Connecticut [35, 36] und in Nordnorwegen [37] mit weiteren Hinweisen in den Literaturverzeichnissen.

Ob sich solche Hexenprozesse auch für Österreich abgehalten wurden, ist mir unbekannt. Überhaupt gibt es nur wenige Berichte über Ergotismus in den österreichischen Kronländern, und zwar aus Böhmen [9, 38–40], Schlesien [41], der Lombardei [42] und Siebenbürgen [10].

## War’s der Roggen oder der Honigtau auf ihm?

In den Gebieten mit der gangränösen Erkrankung wie auch in jenen der Kriebelkrankheit sind diese Leiden stets nach strengen Wintern und nachfolgenden nasskalten Sommern aufgetreten. Die dadurch verursachten schlechten Ernten hatten stets zu Hungersnöten geführt, welche für die armen Gruppen der Bevölkerungen besonders hart gewesen sind. Das nötigte diese Menschen, auch verdorbenes Getreide, insbesondere Roggen, für das Brotbacken zu verwenden. Der Zusammenhang von Ernährung mit schlechtem Getreide und dem Auftreten vom Ergotismus (in der vorerst eingeschränkten, französischen Bedeutung als St.-Antonius-Feuer) und von der Kriebelkrankheit war den betroffenen Bevölkerungen bewusst. Die ärztliche Wissenschaft musste aber erst ihre Standpunkte klären.

Durch den Bericht der französische Société Royale de Médecine vom Jahre 1776 [11] war also die Anschauung einiger damals anerkannter Ärzte vereinigt, dass der Ergotismus (in der französischen Bedeutung) durch das auf Roggenähren wachsendes Mutterkorn verursacht wird, was Tuillier bereits 1630 vermutet hatte [22]. Dafür sprachen auch die Beobachtungen, dass Kranke, die in Asylen oder Hospitälern unter guter Ernährung ihre Symptome verloren hatten, nach Rückkehr in ihre ursprünglichen schlechten Lebensverhältnisse aber wieder erkrankten.

Auch im deutschen Bereich wurde dem Mutterkorn eine krankmachende Wirkung zugesprochen, nämlich für die Entstehung der Kriebelkrankheit. So berichtete Brunner [43] 1695, dass in seiner Gegend, im Schwarzwald, die mit Konvulsionen einhergehende Krankheit durch „granis secalis degenerantia“, also eine Veränderung des Roggenkorns, hervorgerufen wird, wodurch das „Martins-Korn“ [*Mutterkorn*] entsteht.

Nicht unbestritten war die Rolle von Roggen und Mutterkorn. Es gab nämlich auch andere Meinungen, wie z. B. die Verursacher des Ergotismus und der Kriebelkrankheit seien Brandpilze, vegetabile Gifte, starker Schleim, Verengung der Blutgefäße der unteren Extremität, Einflüsse der Luft, Würmer oder Stiche giftiger Insekten [18, 21, 43]. Über Linnés Kreuzblütengewächs Raphanus raphanistrum als vermeintlicher Ergotismus-Erreger ist oben berichtet.

Von manchen Beobachtern wurde der „Honigtau“ als ätiologisches Agens betrachtet. Dieser wird von Insekten (insbes. Blatt- und Schildläuse, Zikaden), die Säfte aus bestimmten Pflanzen, wie auch Roggenähren, als Nahrung aussaugen, aus dem Darmkanal ausgeschieden. Der zucker- und aminosäurenhältige Honigtau wird von anderen Insekten gerne als Nahrung aufgenommen. Wichmann zitiert die Beobachtung von Landsleuten, dass Bienen „verdorbene oder mit Honigthau bedeckte Kornblüthen auf gewissen Aeckern weißlich zu meiden gewußt“ hätten [8]. Sie fragten sich, ob der Honigtau „zu einer Zeit unschädlicher und angenehmer für Insecten wird, als zu einer andern; oder gehöret der Honigthau in das vegetabilische Reich wie der Schimmel, so daß er nicht aus der Luft fällt, sondern sich in feuchtem Erdreiche und Wetter an den Aehren erzeugt, und alsdenn Infusionsthiergen [*Infusionstierchen* *=* *Infusorien*] hervorbringet.“ Nach Taube [6] wollten einige Bienen-Wärter in einem Sommer beobachtet haben, dass „die wenigsten Bienen den Honigtau genossen hätten, die ihn aber doch sammelten, wären nicht zurückgekommen. Bei dauernder trockener und heißer Witterung der Sommermonate hatte es keine Gewitter oder Regengüsse gegeben, die den Honigtau abgespült hätten, sondern dieser wurde an den Aehren eingetrocknet geerntet“ und käme somit in die menschliche Ernährung.

Johann Peter Frank (1745–1821), der mit seinem „System einer vollständigen medicinischen Pollizey“ (1779–1819) die Grundlagen des öffentlichen Gesundheitswesens Österreichs definiert hatte, meinte kurz zur Bedeutung des Honigtaus, dass einige Gelehrte das Mutterkorn von aller Schädlichkeit freisprechen, aber den Honigtau beschuldigen, dass er das Mutterkorn beschmutzt, was öfters Nachteile bringt. Für Frank ist der Honigtau aber nur ein von Blattläusen ausgeschiedener süßer Saft, den Ameisen und Bienen gerne als Nahrung aufnehmen und der außerdem vom Regen abgewaschen wird.

Alle hier genannten Ursachen können wir als falsch in der Besprechung vernachlässigen. Es sind aber die zwei Hauptbeschuldigten, der Roggen und das Mutterkorn, zu besprechen.

## Der Roggen, die Ausnahme unter den Getreiden

In Mesopotamien, 2000 und mehr Jahre v. Chr., waren die primären Getreidearten Gerste und Weizen. Beide Arten sind Selbstbestäuber, bei denen also die meisten Pollenkörner von den Staubblättern auf die Naben von Blüten derselben Ähren gelangen. Dadurch ist ein in der Natur vorkommender genetische Austausch zwischen einzelnen Populationen dieser Getreidearten nur selten zu erwarten. So war es bei solchen Selbstbestäubern möglich, Pflanzen derselben Art mit unterschiedlichen Eigenschaften nahe bei einander ohne Vermischung zu kultivieren und durch Auswahl nützlicher Eigenschaften konstante Kulturpflanzen zu schaffen.

In den Gerste- und Weizenfeldern wuchs ein anderes Süßgras als Unkraut, der Roggen. Dieser ist ein Fremdbestäuber, dessen Pollen durch den Wind vertragen werden. So kommt es zum genetischen Austausch der Roggenpflanzen verschiedener Standorte, seien sie spontan aufgegangen oder von Menschen ausgewählt worden. Alle waren allein dem natürlichen Selektionsdruck unterworfen. Dadurch war es in der Regel nicht möglich, Bestände von Roggenpflanzen mit gemeinsamen vom Menschen selektierten Eigenschaften zu erzeugen, also aus dem primär unerwünschtem Ackerunkraut Roggen durch gezielte Züchtung eine Kulturpflanze zu schaffen, wie es bei Gerste und Weizen bereits frühzeitig gelungen war.

Dieser Zustand veränderte sich nachdem der Roggen als Unkraut über den Vorderen Orient in andere Regionen und schließlich nach Europa gekommen ist. Da dort keine mit ihm verwandten Wildpflanzen vorkamen, fehlte eine genetische Beeinflussung von außen. Im Laufe der Zeit entwickelten sich in den Gerste- und Weizenfeldern auch Roggenpflanzen, deren Körner nach der Reife nicht wie bisher aus den Ähren fielen, sondern in ihnen haften blieben. Nur diese Roggenkörner blieben im Saatgut des folgenden Jahres und wurden mit diesem auf die Ackerflächen ausgesät. Als anspruchslose Getreideart wuchs der Roggen insbesondere auf nährstoffarmen, sandigen Böden und bei Trockenheit und Wärmemangel im Frühjahr und in nasskalten Sommern oft besser als das ursprünglich angebaute Getreide, das größere Ansprüche stellte. So wurde der Roggen durch sein immer stärkeres Auftreten in den Getreidefeldern schließlich zur erwünschten Kulturpflanze.

Von großer Bedeutung für die Zunahme des Roggens hält man auch die Veränderungen der Ernteweise im ersten vorchristlichen Jahrtausend. Die bis dahin verwendeten Steinklingen ermöglichten nur, die mit der Hand festgehaltenen Halme knapp unter den Ähren zu durchtrennen. Dabei wurden wohl Fruchtstände von ungewünschten Unkräutern, wie vom Roggen, gesehen und verworfen. Mit den danach in der Eisenzeit verwendeten metallenen Sicheln konnten die Halme bodennahe geschnitten werden, war nicht nur Getreide, sondern auch Stroh für verschiedene Zwecke lieferte, allerdings sah man nicht mehr so gut Verunreinigungen im Schnittgut.

Aus der späten La-Tène-Zeit, etwa dem letzten vorchristlichen Jahrhundert, mehren sich in Getreidefunden die Körnerzahlen von Roggen, insbesondere in Gegenden mit ärmeren Böden und in Gebirgslagen. Ab dem 8. Jahrhundert und im ganzen Mittelalter entwickelte sich zwischen Seine und Rhein, also im Zentralraum des karolingischen Reichs, eine gesellschaftliche Dynamik. Unter den landwirtschaftlichen Neuerungen, wie etwa der Drei-Felder-Wirtschaft, traten Roggen und Hafer, die da schon als Kulturpflanzen bekannt waren, in den Vordergrund. Mit dem Roggen, der geringe Ansprüche an Temperatur, Niederschläge und Bodenqualität stellte, konnten im Mittelalter Erträge von 500–750 kg pro Hektar erzielt werden, d. i. das Fünffache der Aussaatmengen und weit mehr als mit Weizen zu erreichen war.

Mit Beginn der Neuzeit war Roggen im Großteil Europas das Getreide der Landbevölkerung, die selbst für ihr tägliches Brot sorgen musste. Weizen war dagegen vor allem Handelsware, die in die Städte zur Broterzeugung geliefert wurde.

Die wichtigsten Gebiete, in denen Roggen als Hauptgetreide angebaut wurde, waren Nord- und Mitteldeutschland, die niederländische Geest, Aargau, Wallis, Bayern, Österreich, Böhmen und Schlesien. Es wurde Roggen in weiten Teilen Österreichs und Deutschlands und generell im Norden und Osten Europas zum Hauptgetreide.

[*Die obige Beschreibung der Entwicklung des Roggenanbaus beruht auf den sehr detaillierten Angaben in den Büchern von Michael Mitterauer *[44]*, Rita Kichler & Helmut Reiner *[45]* und Hansjörg Küster *[46, 47].].

In Österreich fanden sich Beweise für den bereits feldmäßigen Roggenanbau im Mittelalter besonders im Wein- und Waldviertel in Michelstetten und Gars-Thunau. In diesem im Mittelalter dicht bewaldeten Teil des nördlichen Niederösterreichs wurde etwa ab dem 10. Jahrhundert im Zuge der vom Donaubereich ausgehenden Kolonisierung des Nordwaldes durch die Reitungen [*Rodungen, Schlägerungen*] „urbares Land“ [*= riuti (althochdt), daher „Reitung“*] geschaffen, das für den Roggenanbau gut geeignet war. Ortsnamen im nördlichen Niederösterreich zeigen den ungeheuren Umfang dieser Arbeiten zur Schaffung von Lebensraum. Es bestehen im nördlichen Niederösterreich Ortsnamen mit Roggen, nämlich Roggenreith und dreimal Roggendorf. Dort weisen auf die Urbarmachung noch heute die amtlichen Namen von 26 Katastralgemeinden mit „‑reith“ und 23 mit „‑schlag“: Edelsreith, Etzelsreith, Frankenreith, Goschenreith, Heinrichsreith, Loibenreith, Mitterreith, Moritzreith, Münichreith, Niederwaltenreith, Oberwaltenreith, Pfaffenreith, Rabesreith, Rapoltenreith, Reith, Roggenreith, Rohrenreith, Sabathenreith, Schirmannsreith, Sieghartsreith, Stopfenreuth, Trabenreith, Wappoltenreith, Wiesenreith, Zabernreith, Zettenreith bzw. Diemschlag, Grafenschlag, Grossheinrichschlag, Haimschlag, Harmanschlag, Hirschenschlag, Immenschlag, Kainrathschlag, Kirchschlag, Kleinpertenschlag, Kleinulrichschlag, Langschlag, Matzlesschlag, Mitterschlag, Oberwindschlag, Ottenschlag, Pfaffenschlag, Rapoltenschlag, Reitzenschlag, Ulrichschlag, Walterschlag, Weikertschlag, Werschenschlag.

**Figure 5:**
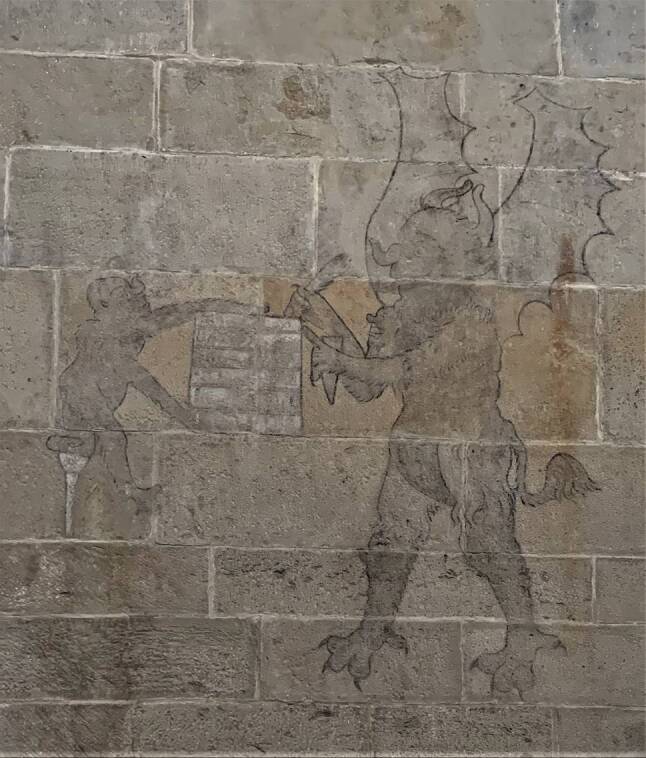

Einen Hinweis auf den frühen Ergotismus in Niederösterreich findet man in der romanischen Kirche in Schöngrabern. An der Innenseite der Südwand des Langhauses ist eine Kohlezeichnung erhalten, deren Entstehung zwischen der ersten Hälfte des 14. und der zweiten Hälfte des 16. Jahrhunderts angenommen wird. Das etwa 3 m hohe Bild zeigt einen geflügelten Teufel mit einem Tintenhorn und einer Feder, dem ein Mann eine Schreibtafel hinhält. Dieser Mann stützt sein fußloses Bein auf eine (hölzerne) Krücke, eine Darstellung, die man auch in vielen Bildern von Bettlern aus der Pestzeit findet (Abb. 5).

Die heutigen Roggenanbaugebiete Österreichs sind das Wein- und Waldviertel als ältestes Anbaugebiet, das westlich anschließende Mühlviertel, das südliche Niederösterreich (Wechselgebiet, Bucklige Welt) mit anschließenden steirischen und burgenländischen Regionen, ferner inneralpine Trockentäler (Inn, Enns, Mur) und der Lungau.

## Eigentlich war’s das Mutterkorn

Für die Betrachtung des Ergotismus bis in unsere Gegenwart ist der Roggen die Voraussetzung, aber nicht die Ursache. Die ist das Mutterkorn, eine Veränderung von einzelnen Körnern vor allem des Roggens. Es ist unter verschiedenen Namen schon lange bekannt als Kornmutter, Mehlmutter, Mutterzapfen, Rockenmutter, Kornzapfen, Martinskorn, Hungerkorn, Brandkorn, Schwarzkorn, Krähenkorn, Hahnensporn, Secale luxurians, Mater secalis, Clavus secalinus, Sphacelus segetum, Ergolaetia abortifaciens und Orga [10] sowie Kornzapfen, Vogelsporn, Todtenkopf, Steinbrand, Seigle ergoté und Grano ghiottono [21]. Interessant ist der sprechende Namen Ergolaetia abortifaciens, heißt dies doch „erfreuliches, abortusmachendes Mutterkorn“.

Der Verursacher des Mutterkorns ist der Schlauchpilz Claviceps purpurea [*Askomyzet, askós, mýkos (griech.)* *=* *Schlauch, Pilz*; *Claviceps (lat.)* *=* *Keulenkopf*]. Er ist ein Parasit gewisser Grasarten. Neben dem Roggen (Secale) als sein Hauptwirt [48] können auch andere Getreide, wie Triticale [*Kreuzung von Roggen und Weizen*], Weizen (Triticum), Gerste (Hordeum), Hirse (Panicum) und Hafer (Avena). Aber auch die zu den Gräsern gehörenden Ackerunkräuter Lolium temulentum (Taumellolch, Schwindelhafer, Schwindel) [6, 21, 28, 39] und Bromus secalinus (Trespe, Roggentrespe, Gänsehafer, Schwingel) [21], welche man ebenfalls zur Mehlerzeugung verwendet hat, sind anfällig. Claviceps befällt auch Futter- und Wildgräser, was für die Haustierhaltung von Bedeutung ist [a*lle betroffenen Grasarten siehe Schubiger* [26]].

Claviceps purpurea durchläuft einen Entwicklungszyklus im Verlaufe eines Jahres. Das Mutterkorn entsteht durch sein Wachstum in den Getreidekörnern und ist seine Dauerform, das Sklerotium [*sklerós (griech.)* *=* *hart*]. Es fällt aus der Ähre zu Boden, wo es überwintert. Zur Zeit der Roggenblüte entwickeln sich aus dem Sklerotium gestielte Köpfchen [*wie Keulen* *=* *clavae*], in denen sich die schlauchartigen Askosporen bilden. Diese werden vom Wind auf die Fruchtknoten des Roggens getragen. Dort bildet sich ein den ganzen Fruchtknoten durchwachsendes Myzel [*Pilzgewebe*] mit zahlreichen Konidien [*Pilzsporen*], die durch Insekten auf weitere Fruchtknoten übertragen werden. Das wachsende Myzel zerstört das ganze Gewebe des befallenen Korns und wächst zu einem harten Gebilde von brauner bis zu dunkelpurpurner Farbe, dem Sklerotium. Dieses ragt schließlich mit bis zu 4 cm Länge aus der Getreideähre heraus. Es bleibt zum Teil in der Ähre und wird mit dem Roggen geerntet, zum Teil fällt es aus der Ähre. Das Sklerotium von Claviceps purpurea bezeichnet man als Mutterkorn oder Secale cornutum [*Secale cereale* *=* *Roggen, cornutus (lat.)* *=* *gehörnt*] [48, 49].

Die Geschichte des Mutterkorns ist wohl noch ein Stück länger als die des Roggens, belegte Hansjörg Küster in seinem persönlichen Brief [50], in dem er berichtete, dass er selbst „Claviceps unter den Getreideresten einer jungsteinzeitlichen Siedlung gefunden hat, die um 3000 vor Chr. bestanden hat. Und in dieser Siedlung wurden nur Gerste, Einkorn und Emmer angebaut. Roggen kam nicht vor. Aber man muss ja bedenken, dass die Getreidearten sehr eng miteinander verwandt sind, so dass sie alle Wirte des Mutterkorns sein können. Vor allem zwischen den Triticum-Arten (Einkorn und Emmer gehören dazu) und Roggen bestehen sehr enge Verwandtschaften“.

## Der „Genuss“ des Mutterkorns

Der formale Vorgang der Aufnahme von Mutterkorn durch die Bevölkerung wurde von mehreren Untersuchern (*z.* *B. Wichmann* [8] *Taube* [6]) genauer beschrieben.

Wichmann berichtete, dass in den Umgebungen von Celle und Gifhorn in Niedersachsen kein einziger Fall von Kriebelkrankheit vor der Roggenernte aufgetreten ist, danach aber ganz allgemein war. Deswegen „war die Ursache entweder in den Nahrungsmitteln oder in der Beschaffenheit der Luft zu suchen. Wenn sie in der Luft gelegen hätte, so würde sie vielleicht allmählich und nicht eben in der Rockenernte ihre Würkung geäussert haben, oder z. E. aus einer Erkältung ec. auch zu einer andern Zeit das Uebel entstanden seyn.“ Für die ursächliche Bedeutung der Ernährung spricht, dass die Landleute in der Heide, einer sandigen, öden Gegend leben, wo außer Buchweizen, Honig und etwas Milch ihre einzige Nahrung der Roggen ist [8].

Da das Mutterkorn wegen seiner Länge aus den Roggenähren hervorragt, fällt es von diesen bei der Bewegung zum Dreschen und bei dessen Einwirkungen leicht aus den Ähren. Das nach dem Dreschen und Abtransport des gedroschenen Korns zurückbleibende „Krümmelkorn“ enthält eine weit größere Menge an Mutterkorn als das abgetragene gedroschene Getreide. Dieses Krümmelkorn wird, besonders in schlechten Jahren, als Erstes zur Nahrung verwendet [8]. Ist die Not besonders drückend, so „säumet der Landmann nicht, von seinem neu eingefahrenen Rocken [*Roggen*] sogleich Brodt zu backen, und da er sich die Zeit nicht geben kann, etwas davon zu dreschen, so sammelt er den Abfall bey dem Einbringen, Krümmelkorn genannt, und führet ihn so gleich zur Mühle. Das Mehl wird eilig verbacken, und das daraus verfertigte Brodt wird, theils aus Neugier, theils aus Nothwendigkeit, warm hinein geschlungen. Diese Gewohnheit war, in der wirthschaftlichen Wohnung, eines zwar kleinen, aber vermögenden Dorfes, namens Lutter [*bei Celle*], so wie in der ganzen Gegend, beybehalten, und die Einwohner hatten schon etliche Tage Brodt vom neuen Krümmelkorn genossen, als die Kriebel-Krankheit daselbst ausbrach“ [6].

Das in Notzeiten verwendete Mehl konnte zu einem Achtel, aber auch bis zur Hälfte aus vermahlenem Mutterkorn bestehen [6, 10].

Für die ursächliche Wirkung der Ernährung spricht auch der Bericht von Taube [6] über den wegen einer heftig wütenden Kriebelkrankheit angeordneten Austausch des neu geernteten Brotgetreides der betroffenen Bevölkerung gegen das von der Königlichen Regierung Niedersachsens ausgegebene einwandfreie. „Die Kranken, welche dieses Glücks theilhaftig wurden, erholten sich nach achttägigem Genuß, so merklich, daß sie sämmtlich ein besseres Aussehen bekamen, und die Heftigkeit der Zufälle verlohren.“ Nach Aufbrauchen des ausgeteilten Getreides mussten die armen Leute wieder ihren eigenen Roggen verwenden und es „stellten sich ihre fürchterlichen Zufälle, und zum Theil noch viel heftigere, ein“.

## Ist das Mutterkorn wirklich giftig?

Nicht alle Zeitgenossen glaubten unbedingt an die Bedeutung des Mutterkorns als giftiges Agens (u. A. [6, 8, 24, 51–54]). Primär bestanden überhaupt Zweifel, ob die gangränöse Erkrankung, der Ergotismus der Franzosen, und die konvulsivische Krankheit, die Kriebelkrankheit, vom selben Erreger erzeugt werden. Conrad Heinrich Fuchs (1803–1855) fragt sich, ob die französische Meinung stimmt, der Ergotismus werde durch Secale cornutum [*Mutterkornpilz*] und die Kriebelkrankheit durch Raphanus Raphanistrum [*Hederich, Ackersenf, Ackerrettich, Wilder Rettich*] erzeugt „oder seien beide die Produkte einer und derselben schädlichen Potenz – des Mutterkornes – unter verschiedenen Verhältnissen, z. B. in verschiedenen Ländern, Klimaten, bei verschiedenen Arten des Säens u. s. w.“ [3].

Johann Ernst Wichmann kommt aus seinen Erfahrungen zum Schluss (1771), wenn er auch „den Kornzapfen [*Mutterkorn*] allein nicht alle Schuld bey der Kriebelkrankheit beylegt, so mögt er sie deswegen doch auch nicht für ganz unschuldig halten, und wenn dieser oder jener einmal beym Spazierengehen [*wegen des angenehmen Geschmackes*] eine geringere Quantität derselben ohne Schaden geniesset, so beweist das höchstens nur, daß sie kein geschwind würkendes Gift sind, welches mancher auch ohne diesen Beweis würde zugegeben haben“ [8].

Johann Georg Model (1711–1775) meinte (1771), wir können mit Wahrscheinlichkeit sagen, „das Mutterkorn ist nicht viel besser, denn schlechter können wir es nicht nennen, als das sogenannte Himmelsmehl [*Lac Lunae solare, ist Mergel-Erde*] oder die Baumrinden, Stroharten u. d. m., die der Arme unter das Mehl mischt, um dieses zu verlängern und nur die leeren Stellen auszufüllen“ [53].

Christian Ehrenfried Eschenbach (1712–1788) glaubte (1771), es bleibt nicht einmal möglich, „dem Mutterkorn die bei dessen Gegenwart sich ereignenden Krankheiten aufzubürden“, man könnte doch nicht die über das Jahr eintretende Krankheit „dem im abgewichenen Herbst mit eingeerndteten und im Brod genossenen Mutterkorn zuschreiben“. Eschenbach „leugnet nur, daß das Mutterkorn die Quelle der schädlichen Folgen gewesen sey.“ Er hält es für unrecht, „mit so seichten Prophezeiungen des Nächsten Gemüthsruhe, in Absicht auf seine Gesundheit, zu stören“ [51].

In seiner „Schutzschrift für das Mutterkorn, als einer angeblichen Ursache der sogenannten Kriebelkrankheit“ (1771) besprach Rudolf Augustin Vogel (1724–1774) zahlreiche Berichte anderer Autoren, die ihn zur Überzeugung verhalfen, dass Mutterkorn kein Gift enthält. Darunter zitierte er auch Charles-Auguste Spielmann (1834–1863), der die Kriebelkrankheit nicht dem Mutterkorn anlastet, sondern eher einer besonderen Beschaffenheit der Luft zuschreibt, die das Korn sowohl wie den menschlichen Körper verdirbt.

Vogel kam zum Schluss, dass „so wenig aber das Mutterkorn an der Kriebelkrankheit Schuld hat, so wenig kann man es für die Ursache einer andern faulichten Krankheit, des trockenen Brandes der äussern Gliedmassen, ansehen, als wofür es von einigen Aerzten auch ausgegeben worden“ ist [54].

Gerhard Matthias Friederich Brawe glaubte „in Ansehung seiner Kranken behaupten zu können, daß der Genuß des sogenannten Mutter-Korns (secale cornutum) schwerlich zu der Entstehung dieser Uebel vieles beygetragen habe. Denn nach denen sehr glaubwürdigen Nachrichten sowol der Herren Beamten“ als auch der Einwohner der Wohnorte seiner Patienten gab es im Jahre der Kriebelkrankheitsfälle 1770 nicht mehr Mutterkorn im Getreide als in den vorausgehenden Jahren. Brawe hält nach seiner Erfahrung Eingeweidewürmer sowie „Empfindlichkeit von Nervenschwäche“ und „Unreinigkeit der ersten Wege“ [*des Darmes*] für die Ursachen der Krankheit (1772) [34].

Johann Taube will nicht „die ganze Schuld und den Ursprung der Kriebelkrankheit auf die vergifteten Kornzapfen“ als Mitschuldige schieben (1782). So muss er „einen gleichen Verdacht auf die mehresten Rockenkörner selbst legen, welche in den Orten in dem Jahre 1770 gewachsen waren, wo die Krankheit herrschte. Unter zwölf Körnern Rocken der angesteckten Gegend, könne man kühnlich den dritten Theil für verdorben angeben“ [6].

Johann Peter Frank behandelte 1792 im Abschnitt der Ernährung noch zwiespältig die ätiologische Bedeutung des Mutterkorns [52]. Einige Berichte über den Genuss von Mutterkorn über viele Jahre ohne Kriebelkrankheit beweisen „aufs höchste, daß das Mutterkorn nicht alljährlich, nicht überall, nicht in jedem auch geringeren Maße schädlich seye. Hier können Umstände eintreten, welche gleiche Wirkungen verhindern. Das älter gewordene Mutterkorn verlieret, nach ziemlich allgemeinen Beobachtungen, vieles von seiner betäubenden Kraft: und der Landmann, welcher nicht gezwungen wird, sogleich das neue Korn zu Brod zu machen, wird keine so merkliche Zufälle davon empfinden. Wer nicht meistens von diesem Brode leben muß, sondern dazwischen verschiedene andere Nahrungsmittel aufzutischen hat, der wird durch diese die Wirkung des nicht allzuhäufigen Mutterkorns ersticken.“ „Die Kriebelkrankheit scheint auf dem Lande, so wie der Schaarbock [*Skorbut*] auf See, alsdann auf schlechte Nahrungsmittel zu entstehen: wenn auf dieser alle vegetabilische Kost, auf dem Lande aber, wenn außer dem von Mutterkorn verderbten Brode, keine animalischen oder andere gesunde Speisen genossen werden können.“ „Die auf ein nasses, der anfänglichen Erzeugung der Kornzapfen günstiges Frühjahr folgenden heißen Sommer können vielleicht das Mutterkorn so austrocknen, daß dieses sein flüchtiges der Gesundheit nachtheiliges Wesen verfließen muß; u. s. w.“

Frank kommt zum Schluss, dass wiewohl das Mutterkorn „in gewissen Jahren unschädlich gewesen seye,“ es nicht selten eine Giftwirkung äußert. Aber der blose Verdacht berechtige schon, das Mutterkorn überall aus der Nahrung zu verbannen, insbesondere da man weiß, dass das Mutterkornmehl keinen Nährwert hat.

## Die ursprüngliche Therapie

Vor der sicheren Feststellung der Bedeutung von Mutterkorn findet man als Therapie ganz verschiedene Maßnahmen, wie Wärme, Räuchern, Baden, Brech- und Abführmittel, „Nervina“ (z. B. Valeriana, Campher, Moschus, Kupfer-Ammonsulfat, Chinarinde, Kalmus, Ingwer) und bestimmte Diäten (z. B. Weißbrot, Fleischbrühen, leichtes Fleisch, weiche Eier, bitteres Bier, kräftigen Wein) [28].

Die anfangs des 19. Jahrhunderts gebräuchlichen Therapien fasst Budetz zusammen [21]. Da alle Beeinträchtigungen ihren Ursprung in einer Überfüllung haben, gilt daher (wenigstens ab Beginn des schlechten Zustands) immer die sofortige Notwendigkeit, entweder das Böse auszuschalten oder die Unschädlichkeit zurückzugeben. Und so ist, ob die Krankheit frisch oder älter ist, die Kur immer mit Evacuantes [*Mittel zur Entleerung im weitesten Sinne*] und sicherlich je nach Unterschied der Fälle ist bald mit Emetica [*Brechmittel*], bald mit Karthatika [*Abführmittel*] anzufangen. Angegeben werden Brechweinstein, Kalomel, Rhabarberwurzel, Jalappa.

Für alle Indikationen befriedigen stimulierende Nervenmittel, seien sie allgemein, tonisierend oder solche, die zu heftigen Reaktionen führen. Es wird eine große Anzahl von Stoffen angegeben.

Als äußere Hilfen werden genannt: verschiedene Bäder, Einreibungen mit zahlreichen Präparaten, Umschläge bei Gliederschmerzen, blasenziehende Mittel bei Stupor [*Gefühllosigkeit*] und beharrlichen Spasmen.

Von einigen Ärzten wurde auch Elektrizität angewandt [55].

Bei Gangrän der Extremitäten wurden Umschläge mir Dekokt von Peru-Rinde oder Bleisalbe angelegt.

Aderlässe, die von manchen Ärzten gelobt, von anderen getadelt wurden, waren nur anzuwenden; wenn Druck im Kopf auf Apoplexie hinwies und Blutungen im Thorax Befürchtungen erregten.

Amputationen, die bei extremen Nekrosen durchgeführt wurden, konnten meist den Tod nicht verhindern.

Das Regimen diaeticum, also die Lebenshaltung, verlangte Leben in sauberer Luft mit genügend Sauerstoff, ausgezeichnete Nahrung, gut verdaulich, vorwiegend animalisch, aber auch mit frischen Vegetabilien, stark verdünnte weiche Getränke, Prisana Hippocratis [*zu Brei gekochte Gerste*], Dekokte von Salep-Wurzel oder Leinsamen usw. oder alkoholische Getränke (bei starker Schwäche) und moderate körperliche Übungen, wenn der Grad der Krankheit es zuließ.

## Die ursprünglichen Verhütungsmaßnahmen

Nach der Feststellung der Ursache des Ergotismus konnten zielführende Maßnahmen zu dessen Vermeidung angegangen werden [21].

Die Behörde sollte die Vermeidung und Entfernung der Ursachen der Kriebelkrankheit durch Überwachung der Lebensmittel sichern. Für Ersteres wurde vorgeschlagen, dass Befall des Getreides durch Secale cornutum [*Mutterkornpilz*], Rubigines [*Rostpilze*] und Ustilagines [*Brandpilze*] sowie durch unnütze und schädliche Pflanzen festgestellt und durch sorgfältigen Ackerbau gehemmt werden sollen.

Das noch nicht gut getrocknete Getreide ist in geräumigen, belüfteten und begehbaren Scheunen aufzubewahren und durch häufigeres Belüften vor dem Verderben zu bewahren. Wenn dies nicht beachtet wird, befällt der Kornwurm das Getreide.

Von verschiedenen Samen und den genannten Pilzen wird das Getreide in bewährter Weise gereinigt, bevor es zu den Mühlen und Bäckereien gebracht wird. Gereinigt werden kann es durch Siebe oder Waschen; das Mutterkorn und die durch Rostpilze befallenen Körner schwimmen meistens auf der Wasseroberfläche.

Schädliches, verunreinigtes Getreide kommt oft vor. Außer bei großer Ernte, wird es bis zum Gebrauch gut getrocknet. Dem daraus erzeugten Mehl wird vor der Übergabe an den Bäcker gutes Mehl zugemischt. Das geschieht auch bei Mehl aus gutem Getreide, das jedoch in Kästen und Fässern länger der Feuchtigkeit ausgesetzt war und dabei verdorben ist.

Die Müller müssen aufpassen, dass sie nicht schlecht fermentiertes, klebriges, aus nicht genug gemahlenem oder schleimigem oder anders verdorbenem Mehl gemachtes Brot verkaufen.

Zur Vermehrung der zu verkaufenden Mengen von Mehl wurde von Betrügern das Mehl durch Zusatz von entkalktem Sand, Tonerde oder Gips verfälscht, dadurch unbrauchbar und als höchst schädlich nicht zu verwenden. Im Gegensatz dazu nennt Johann Peter Frank [52] weitere unschädliche Mehlstreckungsmittel, die z. T. Nährwert besitzen, aber z. T. nur das Volumen im Verdauungstrakt erhöhen: Hülsenfrüchte, Wurzeln, Eicheln, Bucheckern, Kastanien, Kürbisse, Erdäpfel, Rüben und noch etliche andere pflanzliche Produkte.

Auf Grund seiner Beobachtung des Auftretens der Kriebelkrankheit nur in jenen Ortschaften, wo die Leute sofort nach der Ernte gedroschen und mit diesem Korn Brot gebacken haben, gestattete sich Johann Anton Scrinci im Jahre 1737 „in gehorsamstem Respekt, vermög seines guten Gewissens einer Hochlöbl. Königl. Stadthalterey [*in Prag*] pro remedio diese graßirende Seuche abzuschneiden“, Folgendes zu „offeriren und submittiren“: 1.) Einziges Mittel zur Behebung der Seuche ist, den Leuten vom Unkraut befreites, gutes Brot zu geben, 2.) Dieses muss gut ausgebacken und nicht mehr heiß sein, 3.) Das liebe Brot darf nicht „mit dem Turmkraut [*auch Thurmsenf, Thurmkohl, Arabis glabra, ein Kreuzblütler*] in der Gersten und dem Schwindel-Haber [*Lolium temulentum*] oder auch mit dem schlechten Schroth“ [*in dem sich das Mutterkorn ansammelt*] gemischt werden, 4.) Die Dorfbäcker müssen verpflichtet werden, dass sie aus diesem verbotenen Mehl kein Brot backen und den armen Leuten verkaufen, 5.) Die Müller dürfen aus solchem vergifteten Schrot kein Mehl machen und verkaufen, 6.)„Brauchten wohl ein Vorrath von gutem Getrayd die armen Leute den grossen Hunger zu stillen“ [40].

## Die Mutterkornalkaloide

Wässrige Mutterkornextrakte wurden bereits im Mittelalter in der Geburtshilfe eingesetzt [*Ergolaetia abortifaciens*]. Ab der ersten Hälfte des 20. Jahrhunderts wurden aus dem Mutterkorn stammende Alkaloide chemisch und pharmakologisch intensiv untersucht. Dabei stieß der Schweizer Chemiker Albert Hofmann (1906–2008) bei seinen umfänglichen chemischen Untersuchungen im Betrieb von Arthur Stoll (1887–1971) auf therapeutisch wirksame Ergotalkaloide und bei einer ihrer Synthesen 1943 auf das Rauschgift Lysergsäurediethylamid, das Suchtgift LSD [56].

Die Mutterkorn- oder Ergotalkaloide beruhen auf dem tetrazyklischen Ergolinring-System und werden in vier Hauptgruppen gegliedert: Clavin‑, Lysergsäure‑, Ergopeptin- und Ergopeptam-Alkaloide. Die wichtigsten Ergotalkaloide sind Ergometrin, Ergosin, Ergocristin, Ergocryptin und Ergocornin sowie deren Epimere [*Ergometrinin und weitere -ine*]. Bei der Untersuchung von Roggen, Gerste, Weizen, Hafer und Tritikale waren Ergotamin, Ergosin und Ergocristin die vorherrschend beachteten Alkaloide [57].

Die Ergotalkaloide sind relativ unstabil. Im unversehrten Mutterkorn nimmt die Wirksamkeit nur langsam ab, während sie sich nach dem Mahlprozess durch veränderte Oberflächen-Volumen-Verhältnisse ziemlich rasch verringert [58]. Der Backprozess soll nach der „Bundesanstalt für Getreide‑, Kartoffel- und Fettforschung“, BRD, den Gesamtalkaloidgehalt um etwa 50 % reduzieren [59].

Den Gehalt des [*wohl frischen (?)*] Mutterkorns an Ergotalkaloiden zitiert das „Bundesinstitut für Risikobewertung“, Berlin, mit 0,01–0,5 % [59]. In der Trockensubstanz fand Knapp vom „Bayerischen Landesamt für Gesundheit und Lebensmittelsicherheit“ 0,1–1 % Ergotalkaloide [60].

Die Ergotalkaloide haben vielfältige toxische Eigenschaften: kanzerogene, mutagene, teratogene, östrogene, hämorrhagische Wirkung, Immuno‑, Nephro‑, Hepato‑, Dermato‑, Zyto- und Neurotoxizität [61].

Bei der akuten Toxizität findet man zunächst unspezifische Symptome wie Tachykardie, Durstgefühl, Erbrechen, Schwindel, Durchfall, Blutdrucksteigerung, Kopfschmerzen, Pupillenerweiterung, Wechsel von Kälte- und Hitzegefühl, Parästhesien, Gefühllosigkeit und Krämpfe der Extremitäten, Kontraktionen und Blutungen des graviden Uterus und Abortus; der Tod tritt durch Atemlähmung oder Kreislaufversagen ein [59, 62].

Die chronische Vergiftung beginnt mit Durchfall, Erbrechen, Schwindel, Taubheit und Schmerzen der Gliedmaßen oder der Körperoberfläche. Daraus entwickeln sich der Ergotismus gangraenosus oder der Ergotismus convulsivus [59, 62].

## Das Problem Mutterkorn ist nicht gelöst

Die Fachzeitschrift „Land & Forst“ ermahnte 2015 ihre fachlichen Leser mit dem Artikel „Mutterkorn ernst nehmen“ [63]. Und im Juni 2020 wies sie unter der Überschrift „Problem Mutterkorn ist nicht gelöst“ erneut auf die Wichtigkeit der Kenntnis und Verhütung des Ergotismus hin [64].

Das „Bundesinstitut für Risikobewertung“, Berlin, hatte in seiner „Stellungnahme vom 22. Januar 2004“ erklärt, dass der Verzehr von Roggenmehlen, deren Gesamtgehalte an Ergotalkaloiden zwischen 2308 und 3138 µg/kg liegen, unerwünschte Wirkungen haben [59]. „Aus Vorsorgegründen muss dringend vor dem Verzehr dieser Mehle abgeraten werden. Dies gilt insbesondere für Schwangere bzw. ungeborene Kinder und gestillte Säuglinge“. Dieser Aussage lag die Annahme eines täglichen Verbrauchs von 250 g Roggenmehl zugrunde.

Aus dem Jahre 2007 liegen Untersuchungsergebnisse des „Bayerischen Landesamtes für Gesundheit und Lebensmittelsicherheit“ (LGL) vor [65]. Von 204 aus Getreiden und Getreideprodukten gezogenen Proben waren Ergotalkaloide bei Roggen in 55 %, bei Backwaren mit Roggenanteil in 56 % und bei Roggenbrot in 67 % nachweisbar, fanden sich aber z. T. auch bei Weizen und Dinkel. Davon wurden als gesundheitsschädlich je eine Probe Roggenkörner (= 6 % von 18 Proben), Roggenmehl (= 3 % v. 40), Weizenkörner (= 100 % v. 1) und Weizenmehl (= 3 % v. 33) eingestuft. „Zehn weitere Erzeugnisse zeigten im Vergleich zu ähnlichen Produkten auffällig hohe Gehalte, die das LGL aber aufgrund der nachgewiesenen Mengen noch nicht als gesundheitsschädlich beurteilte“.

Einen Überblick über in den Jahren 2011–2016 in 15 verschiedenen europäischen Ländern festgestellten Gehalte an Ergotalkaloiden bieten die Erhebungen der „Europäischen Behörde für Lebensmittelsicherheit“ (EFSA, European Food Safety Authority): Roggenmahlprodukte 198–239 µg/kg, Weizen-Roggen-Mischbrote 33–82 µg/kg, Roggenflocken 35–83 µg/kg [57].

Die schwankenden standort-, sorten- und jahresabhängigen Unterschiede im Befall durch die Mutterkornsklerotien gelten auch für deren unterschiedliche Alkaloidkonzentrationen [56, 60]. Aber es ist auch zu bedenken, dass die Sklerotienmenge und der Toxingehalt in mutterkornkontaminiertem Getreide nicht korrelieren müssen, z. B. durch anhaftenden Mutterkornstaub an der Oberfläche intakter Roggenkörner [66]. Als tödliche Dosis für Erwachsene werden im Allgemeinen 5–10 g Mutterkornsklerotien angenommen [60].

Wegen der also noch immer bestehenden Gesundheitsgefährdung durch Mutterkorn wurde die diesbezüglich im Jahre 2006 erlassene Verordnung der Europäischen Union durch die Verordnung (EU) Nr. 2021/1399 über „Höchstgehalte an Mutterkorn-Sklerotien und Ergotalkaloiden in bestimmten Lebensmitteln“ [67] geändert. Es gelten also ab 1. Jänner 2022 folgende Höchstgehalte:

- Höchstgehalte von Mutterkorn-Sklerotien
  - unverarbeitetes Getreide außer Mais, Roggen und Reis: 0,2 g/kg
  - unverarbeiteter Roggen 0,5 g/kg (ab 01.07.2024: 0,2 g/kg)
- Höchstgehalte von Ergotalkaloiden
  - Mahlerzeugnisse aus Gerste, Weizen, Dinkel und Hafer mit einem Aschegehalt von weniger als 900 mg/100 g: 100 µg/kg (ab 01.07.2024: 50 µg/kg)
  - Mahlerzeugnisse aus Gerste, Weizen, Dinkel und Hafer mit einem Aschegehalt von mindestens 900 mg/100 g: 150 µg/kg,
  - Gersten‑, Weizen‑, Dinkel- und Haferkörner, die für den Endverbraucher in Verkehr gebracht werden: 150 µg/kg.
  - Roggenmahlerzeugnisse aus Roggen, der für den Endverbraucher in Verkehr gebracht wird: 500 µg/kg (ab 01.07.2024: 250 µg/kg).
  - Weizengluten: 400 µg/kg
  - Getreidebeikost für Säuglinge und Kleinkinder: 20 µg/kg.

[*Höherer Aschegehalt von Mahlerzeugnissen zeigt deren höheren Kleie-Anteil an und somit einen höheren Gehalt an Ergotalkaloiden, da die Kleie den Staub von Mutterkorn-Sklerotien adsorbiert*.].

Bei dem „Trend zur alternativen Lebensweise mit einer „natürlichen“ Ernährung und der damit verbundenen „Hausmüllerei und -bäckerei“ besteht wieder die Gefahr, dass Mutterkorn vor dem Schroten nicht entfernt wird“, wenn „naturnah belassenes“ Getreide z. B. zur Bereitung selbstgemachter Müslis verwendet wird [68]. Das „Bayerische Landesamt für Gesundheit und Lebensmittelsicherheit“ [60] warnte im Jahre 2021 ausdrücklich „vor dem Verzehr von ungereinigtem Getreide, da dann chronische und akute Vergiftungen nicht auszuschließen sind. Dennoch finden sich auch heute noch Getreide und Getreideerzeugnisse, die teilweise sehr hohe Gehalte an Ergotalkaloiden aufzeigen und bei denen eine gesundheitsschädliche Wirkung nicht ausgeschlossen werden kann.“

Die Lebensmittel-Hygiene muss sich auch mit der Frage des sogenannten „Carry-over“ bei tierischen Lebensmitteln befassen, also der Übertragung der Ergotalkaloide aus Fleisch, tierischen Organen, Milch und Eiern von Tieren, die Mutterkorn mit ihrem Futter aufgenommen haben.

Die prinzipielle Schädlichkeit des Mutterkorns für die Haustiere selbst haben viele Landleute schon im 18. Jahrhundert beobachtet, die zur Roggenerntezeit sahen wie „hin und wieder plötzlich einige Thiere als Schweine ec. gefallen sind, und Hühner von dem Wasser, worinn das unreine Korn gewaschen worden, etwa auch von den darinn zurückgebliebenen, verdorbenen Körnern“ „Convulsionen bekommen, die ihnen zwar nicht tödtlich gewesen, aber doch die Flügel gelähmet haben“. Als Augenzeugen beschrieben z. B. Wichmann [8] und Taube [6], dass Schweine an den Hinterbeinen aus unbekannten Ursachen halb gelähmt, sich auf den Scheunen „herumgeschleppt und so getaumelt haben wie Kinder im Krampfanfall“. Nicht seien jedoch Pferde, Hornvieh und Hunde erkrankt gewesen.

Durch Fressen von Gräsern mit Mutterkornbesatz auf Weiden, Verfütterung von mit Mutterkorn belasteter Silage und von Futtermitteln können die Allgemeinzustände der Tiere beeinträchtigt werden und es entstehen Hyperthermie, Krämpfe, Gangrän der Extremitäten, reduzierte Fertilität und auch der Tod [61]. Bei Kühen kann die Milchproduktion vermindert bis ganz unterbrochen werden; die Mutterkornalkaloide gehen aber nicht in die Milch über [59].

Auf Grund der in Deutschland in Futtermitteln der Jahre 2011–2016 gefundenen Ergotalkaloide, die zwar einen negativen Einfluss auf die Tierproduktion ausüben können, rechnen jedoch Gareis und Wolf von der „Bundesanstalt für Fleischforschung“ und der „Bundesanstalt für Getreide‑, Kartoffel- und Fettforschung“ (beide BRD) nach damaligen Kenntnissen nicht mit einer Belastung des Menschen durch Lebensmittel tierischer Herkunft [61].

Neuerdings untersuchten Zebeli und seine Arbeitsgruppe von April bis Oktober 2019 die Weiden von 18 Milchbetrieben in Nieder- und Oberösterreich und in der Steiermark. Die Werte von Mutterkornalkaloiden waren generell niedrig, aber es gab durchaus auch Weiden, auf denen insbesondere in heißen Jahreszeiten Mengen von bis zu 500 µg/kg Trockenmasse gefunden wurden. Zebeli befürchtet jedenfalls zunehmende Konzentrationen in heißen Sommermonaten, wenn man bedenkt, dass die Sommer immer heißer und trockener werden [69, 70].

Wie für die menschliche Ernährung gibt es auch Höchstwerte für „unerwünschte Stoffe, die in Futtermitteln enthalten sind und sich auf die tierische und menschliche Gesundheit nachteilig auswirken können“. Der in der österreichischen „Futtermittelverordnung 2010“ in der derzeit gültigen Fassung aus 2017 angegebene Höchstgehalt an Mutterkorn beträgt 1000 mg/kg der ungemahlenes Getreide enthaltenden Futtermittel (bezogen auf Futtermittel mit einem Feuchtigkeitsgehalt von 12 %) [71].

Neben der alimentären Mutterkornvergiftung darf auch ein arbeitshygienisches Problem nicht übersehen werden, die respiratorische Aufnahme von Ergotalkaloiden. Berufsbedingt kann Staub mit Sklerotienteilchen eingeatmet werden. Dies ist sowohl in der Landwirtschaft als auch in der getreideverarbeitenden Industrie möglich. Als ein Beispiel sei hier der Fall eines 42-jährigen Landwirtes genannt, der einer chronischen Exposition gegenüber Staub beim Mahlen stark mutterkornhaltigen Roggens ausgesetzt war [72]. Die sehr schweren Stenosierungen der Unterschenkel- und Fußarterien konnten in viermonatiger Therapie nahezu vollständig behoben werden. Das nur langsame Absinken des Ergotaminspiegels ließ auf eine Toxinakkumulation in einem unbekannten Depot im Körper mit verzögerter Abgabe ins Blut schließen.

## Die technische Minimierung der Gefahr durch Mutterkorn

Die Bekämpfung der Mutterkornvergiftung muss bereits in der landwirtschaftlichen Produktion durch Verringerung des Mutterkornbefalls einsetzen. Dies sind züchterische Auswahl (gering anfällige Sorten bei jährlichem Wechsel) und pflanzenbauliche Maßnahmen wie Standortauswahl, Freihalten der Feldraine von mutterkorninfizierten Gräsern, wechselnde Fruchtfolgen und Einpflügen der Sklerotien unter 25 cm Tiefe. Trotzdem ist der Befall von Getreide durch Claviceps purpurea nicht gänzlich zu verhindern.

Das geerntete Getreide muss vor dem Mahlen behandelt werden. Es sind also einerseits alle gröberen Fremdstoffe, wie Steine, andere Getreidekörner, Unkrautsamen und Mutterkorn zu entfernen und andererseits die Getreideoberflächen von losen Schalen, Staub, Insektenfragmenten und Ausscheidungen von Nagetieren zu reinigen. Die Sortierung erreichen die auf verschiedenen Eigenschaften des Ernteguts beruhenden Geräte und Maschinen, die Reinigung der Körneroberflächen übernehmen Scheuermaschinen [45, 66, 71].

**Figure 6:**
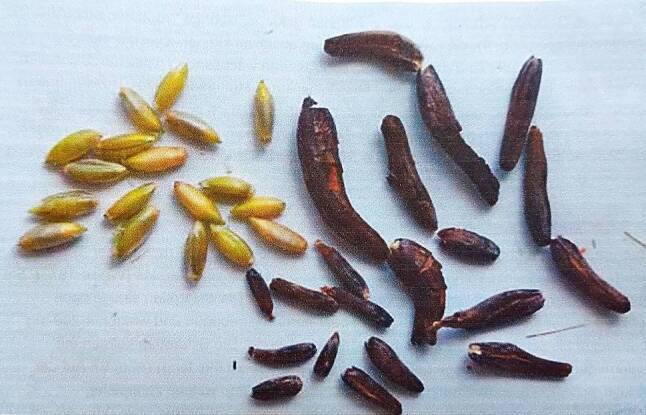

Die Abtrennung von Mutterkorn ist besonders schwierig, da dieses in verschiedenen Größen und Formen und auch als Bruchstücke im Getreide vorkommt (Abb. 6). Eine weitgehende Entfernung von Sklerotien ist nur durch die Kombination verschiedener Reinigungssysteme und -prinzipien möglich. In der Sortierung werden über die Siebung Sklerotien, Sklerotienbruchstücke und Verunreinigungen ausgesondert, die kleiner oder größer als ein Getreidekorn sind. Nach deren Form und Größe trennen die Trieure verschiedener Bauarten die einzelnen Fraktionen [*trier (franz.)* *=* *sortieren, wie bei der Triage von Patienten*]. Den Unterschied der spezifischen Gewichte setzen Tischausleser und Leichtausleser ein.

Die modernen opto-elektronischen Farbsortiergeräte erkennen die dunklere Farbe des Mutterkorns und seiner Bruchstücke zur Unterscheidung vom Getreide. In den Farbsortierern werden die Körner einzeln beleuchtet und dabei an Hochgeschwindigkeitskameras vorbeigeleitet. Bei Erkennung einer dunkleren Farbe wird ein Druckluftstoß ausgelöst, der das dunklere Gebilde ausbläst.

Durch die heutigen Reinigungsverfahren kann ein Mutterkornbesatz des Ernteguts von einigen Prozenten auf 0,05 % gesenkt werden. Trotzdem zurückbleibende Mutterkörner und deren Bruchstücke werden gemeinsam mit dem Korn vermahlen. Sie kommen aber aufgrund unterschiedlicher Eigenschaften ihrer Strukturen nicht gleichmäßig in jede der einzelnen Mahlfraktionen.

Münzing von der „Bundesforschungsanstalt für Ernährung und Lebensmittel“ in Detmold, BRD, betonte 2006 aufgrund der bundesweiten Befallswerte, „dass Roggenverarbeiter selbst in unproblematischen Erntejahren ein mutterkornorientiertes Risikomanagement pflegen müssen, selbst dann, wenn die meisten Druschgut-Anlieferungen unproblematisch erscheinen“. „Solange die zu erwartenden Erfolge der Resistenzzüchtung bei Roggen ausbleiben, wird das Mutterkornrisiko weiterhin beachtet werden müssen“ [66].

Diese Aussage und der zunehmende Trend zur „gesunden naturnahen“ Ernährung mit vom Erzeuger direkt gekauften Getreiden für die eigene Herstellung von z. B. Müslis müssen die Ärzteschaft an die noch immer notwendige Kenntnis des Ergotismus erinnern.

## Schlussfolgerung

Die noch im 19. Jahrhundert viele Todesopfer fordernde lokal auftretende Massenerkrankung an Mutterkornvergiftung hat durch umfangreiche landwirtschaftliche und technische Maßnahmen ihre Schrecken verloren. Da der Erzeuger des Mutterkorns, der Pilz Claviceps purpurea, noch immer in der Natur um uns herum vorkommt, sind die von der Europäischen Union vorgegebenen Höchstwerte in bestimmten für die menschliche und tierische Ernährung benötigten Getreideprodukten weiterhin einzuhalten und dies muss weiterhin kontrolliert werden. Die Ärzteschaft soll bei Feststellung von für Ergotismus möglichen Symptomen anamnestisch die Essgewohnheiten („naturnahe Ernährung“?) und allfällige berufliche Expositionen von den Patienten erfragen.
